# Surface Oxidation of TiNiSn (Half-Heusler) Alloy by Oxygen and Water Vapor

**DOI:** 10.3390/ma11112296

**Published:** 2018-11-15

**Authors:** Oshrat Appel, Shai Cohen, Ofer Beeri, Noah Shamir, Yaniv Gelbstein, Shimon Zalkind

**Affiliations:** 1 Department of Materials Engineering, Ben-Gurion University of the Negev, POB 653, Beer-Sheva 84105, Israel; noah.shamir@gmail.com (N.S.); yanivge@bgu.ac.il (Y.G.); 2 Nuclear Research Centre-Negev, POB 9001, Beer-Sheva 84190, Israel; scking1@gmail.com (S.C.); ofer.beeri@gmail.com (O.B.)

**Keywords:** TiNiSn, half-Heusler, thermoelectric, segregation, surface oxidation, oxygen, water vapor, XPS

## Abstract

TiNiSn-based half-Heusler semiconducting compounds have the highest potential as *n*-type thermoelectric materials for the use at elevated temperatures. In order to use these compounds in a thermoelectric module, it is crucial to examine their behaviour at a working temperature (approximately 1000 K) under oxygen and a humid atmosphere. Auger electron spectroscopy (AES) and X-ray photoelectron spectroscopy (XPS) were utilized to study the surface composition and oxidation of the TiNiSn alloy at elevated temperatures. It was found that during heating in vacuum, Sn segregates to the surface. Exposing the alloy to oxygen at room temperature will cause surface oxidation of Ti to TiO_2_ and Ti_2_O_3_ and some minor oxidation of Sn. Oxidation at 1000 K induces Ti segregation to the surface, creating a titanium oxide layer composed of mainly TiO_2_ as well as Ti_2_O_3_ and TiO. Water vapor was found to be a weaker oxidative gas medium compared to oxygen.

## 1. Introduction

Thermoelectric materials have a great potential to convert waste heat into electricity and are being widely investigated, mainly for decreasing the dependence on conventional fossil fuels and to reduce harmful emissions [1]. Different classes of materials are being applied for different applications, depending on their actual working temperature. For high temperature applications (up to 1000 K), half-Heusler (HH) compounds are thermoelectrically attractive intermetallic semiconducting compounds, exhibiting high Seebeck coefficient and low electrical resistivity values along with superior mechanical properties, non-toxic elements and low cost of the consisting elements [2,3,4]. The HH semiconducting compounds with 18 valence electrons are based on the general formula of XYZ (X, Y = transition metals or rare-earth metals and Z = main group element). The compounds crystallize in the cubic MgAgAs-type structure ($F\bar{4}3m$) and can be derived from the tetrahedral ZnS-type structure by filling the octahedral lattice sites [4]. One of the most investigated HH compounds is the *n*-type TiNiSn-based family, which is attractive due to its relatively high thermoelectric figure of merit (ZT) [5,6,7,8,9,10,11,12,13,14,15]. For practical applications, it is crucial to take into account other considerations, such as the environmental stability at working conditions. Although numerous studies have been conducted regarding alloy metallurgical and transport properties, only few studies can be found on the corrosion behaviour and environmental stability, especially at high operation temperatures, which are a key factor for the commercial utilization of the alloys. Bankina et al. [16] studied the oxidation of TiNiSn powder upon heating in air by X-ray diffraction and found that the oxidation proceeds mainly by the formation of lower titanium and tin oxides accompanied by the release of metallic tin and formation of nickel enriched intermetallics. Galazka et al. [17] utilized thermogravimetric analysis (TGA) measurements on Ti_0.33_Hf_0.33_Zr_0.33_NiSn compound, to follow the surface oxidation. They found that oxidation started at ~545 K and a significantly mass change was measured above 673 K. The oxidation rate was initiated by a log (t) dependence, indicating a kinetically controlled first stage, and was followed by a parabolic dependence, indicating that diffusion through the formed oxide becomes the rate determining step. Berche and Juned [18] studied the oxidation behaviour of TiNiSn by ab initio methods. Their calculations indicate that oxygen, incorporated into the matrix, partially decompose the TiNiSn phase into Ti_2_O_3_ and nickel stannides, which may deteriorate the global thermoelectrical performance. As of now, no surface analysis of the oxidation of the TiNiSn HH compound (and similar HH alloys) have been found.

In the present work, temperature effects on the surface composition (segregation) were studied with a major focus on the oxidation of the TiNiSn (HH) alloy at room temperature (RT) and 1000 K, utilizing Auger electron spectroscopy (AES) and X-ray photoelectron spectroscopy (XPS).

## 2. Experimental

**Figure 1:**
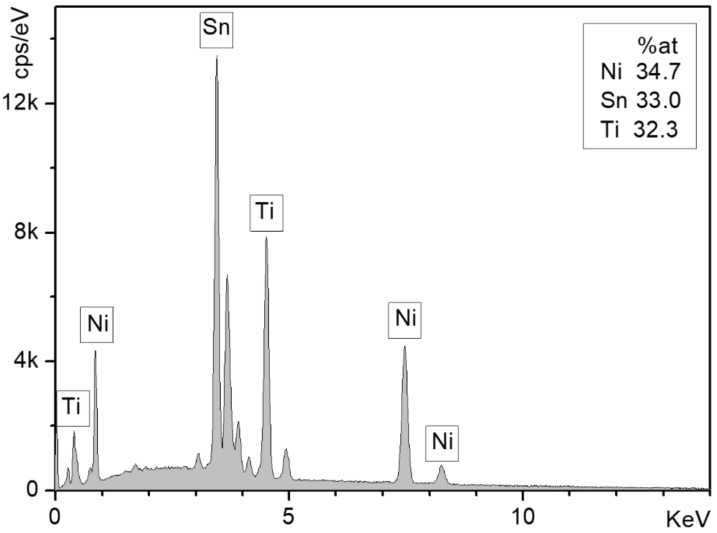
EDS spectrum of the sample surface.

Arc melting under an Ar atmosphere was used to prepare the TiNiSn alloy from high-purity Ti (99.99%), Ni (99.999%) and Sn (99.999%). The alloy was re-melted 5 times and the composition and homogeneity of the alloy were verified by scanning electron microscopy (SEM, JSM 5600, JEOL Ltd., Tokyo, Japan) and energy dispersive spectroscopy (EDS, Thermo Fisher Scientific, Waltham, MA, USA) (Figure 1).

An 8 mm diameter, 1 mm thick coupon of the alloy was gradually polished using diamond paste, down to 1 µm roughness, washed in distilled water and ethanol. The sample was attached to two Ta wires, which enabled heating by driving an electric current through them. The sample’s temperature was monitored by a chromel–alumel thermocouple, spot-welded to the sample edge.

The experiments were performed in ultra-high-vacuum (UHV) systems, pumped by turbo-molecular and titanium sublimation pumps to a base pressure of ~2 × 10^−10^ Torr, monitored by a Bayard-Alpert type ionization gauge and a quadrupole residual gas analyser (RGA, SRS 100, Stanford Research Systems, Sunnyvale, CA, USA). The experiments were performed utilizing standard surface analysis instrumentation for AES (PHI, 15-255G analyzer, Chigasaki, Japan) and XPS (ESCALAB 250, Thermo Fisher Scientific, Waltham, MA, USA) and a differentially pumped rastered Ar^+^ gun for surface cleaning. The XPS measurements reported here were performed using an Al Kα source and spectrometer pass energy at 50 eV. The Au 4f_7/2_ peak at binding energy (BE) of 84 eV and Sn 3d_5/2_ at 485 eV, taken from clean pure reference samples, at similar parameters as of the alloy, were used to verify the energy calibration.

The oxidation experiments were performed by backfilling the vacuum chamber with O_2_ (99.999%) or with distilled H_2_O vapor (further purified from dissolved gases by successive freeze-pump-thaw cycles), via leak valves, to a pressure of 1 × 10^−6^ Torr. The Bayard–Alpert ionization gauge was calibrated for N_2_ and since the ionization cross section of O_2_ and H_2_O are close to unity (1.01 and 1.1, respectively), no further calibration was done. The exposure is given in Langmuir (1L = 1 × 10^−6^ Torr × 1 s.)

The spectra were analysed using Casa XPS software (2.3.15, Casa Software Ltd., Cheshire SK9 6BN, Devon, UK). The XPS data analysis was accomplished using Shirley background and the constrains on energy locations of the different oxidation states and split-orbit area ratio are detailed in [19,20,21,22,23]. A mixture of Gaussian–Lorentzian line shape GL (30) was used for the oxides fitting while for the metallic core lines a slightly asymmetric shape in the form of LA (α,β,m) was used. The estimated statistical error of the XPS measurements combined with the peaks fitting is less than 10%.

## 3. Results and Discussion

### 3.1. Surface Characterization and Segregation

**Figure 2:**
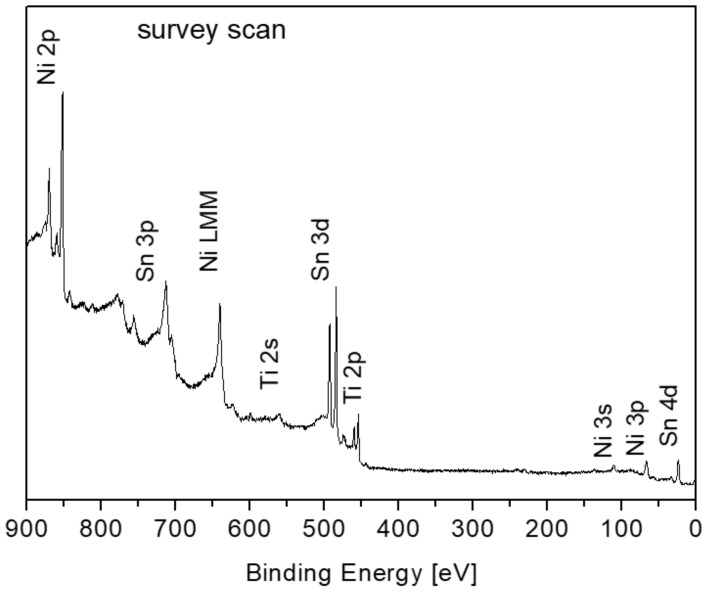

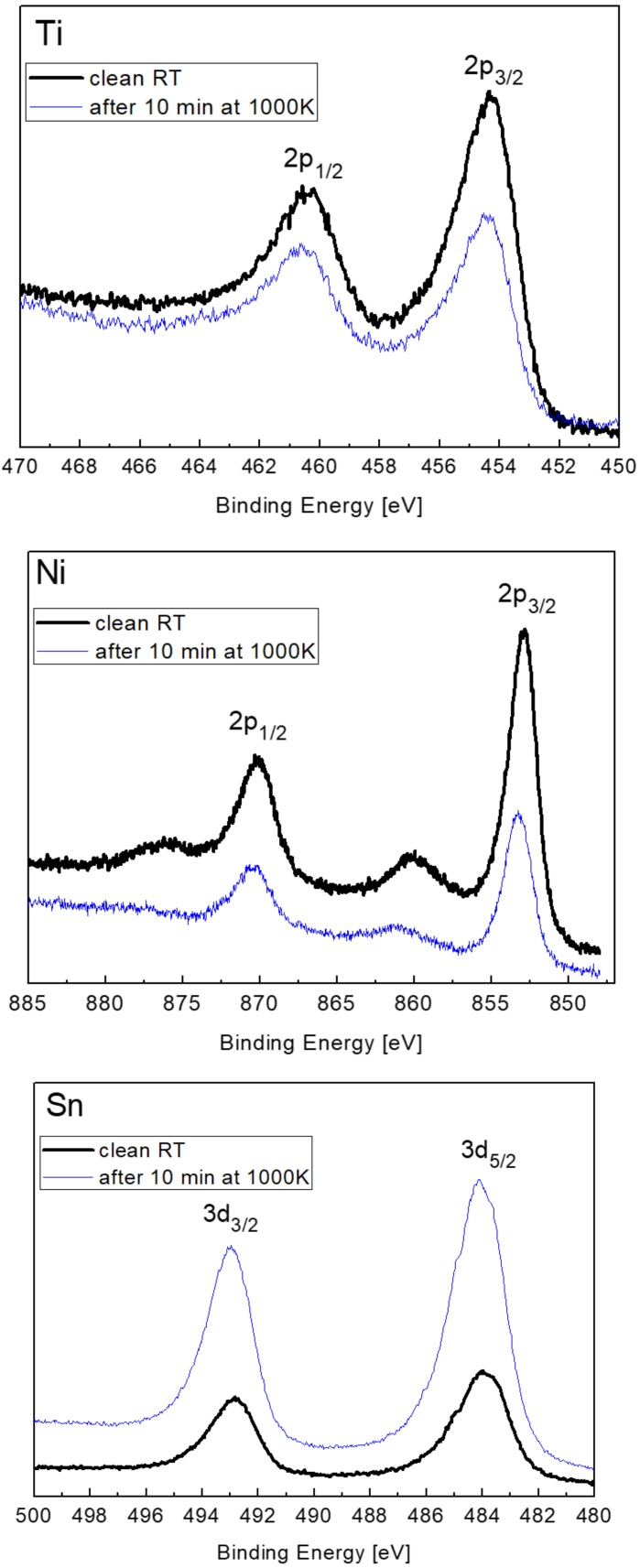
XPS survey scan of the alloy at room temperature and high-resolution spectra of the alloy components at room temperature and after heating (in vacuum) to 1000 K for 10 min (in arbitrary units).

A survey XPS spectrum of the clean alloy at room temperature (RT) and high-resolution spectra of the alloy components at RT and after heating (in vacuum) to 1000 K are shown in Figure 2. No measurable oxygen or carbon contamination could be observed after the sputter cleaning. It can be seen that upon heating a few minutes at 1000 K the tin signal increases and the titanium and the nickel signals are attenuated, implying that Sn segregates to the surface. Segregation is usually driven by bulk elastic strain energy relief of the lattice or by the tendency to reduce the surface energy [24]. Looking at the surface energy of the components shows that Sn has a considerably lower surface energy (~0.7 J/m^2^) compared to Ti (~2 J/m^2^) and Ni (~2.4 J/m^2^) [25] and therefore it is expected to segregate to the surface upon heating.

**Figure 3:**
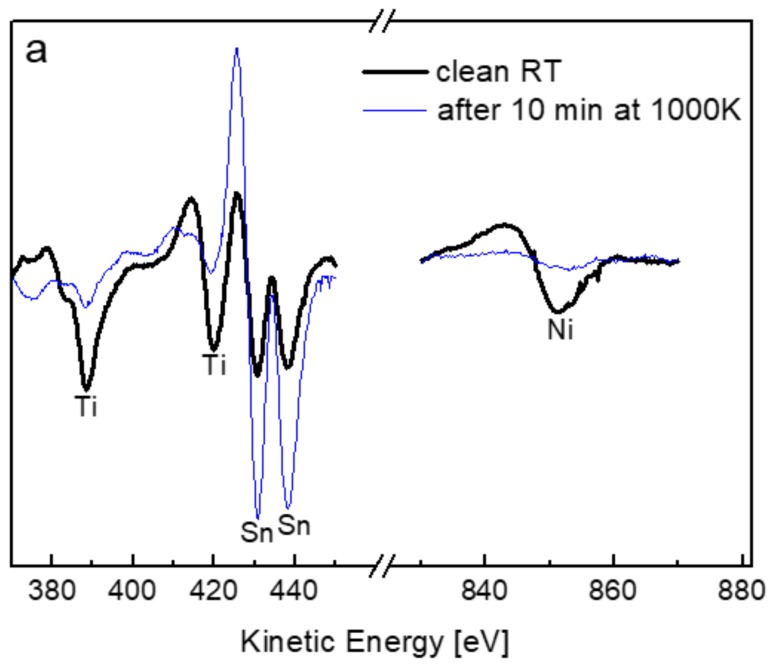

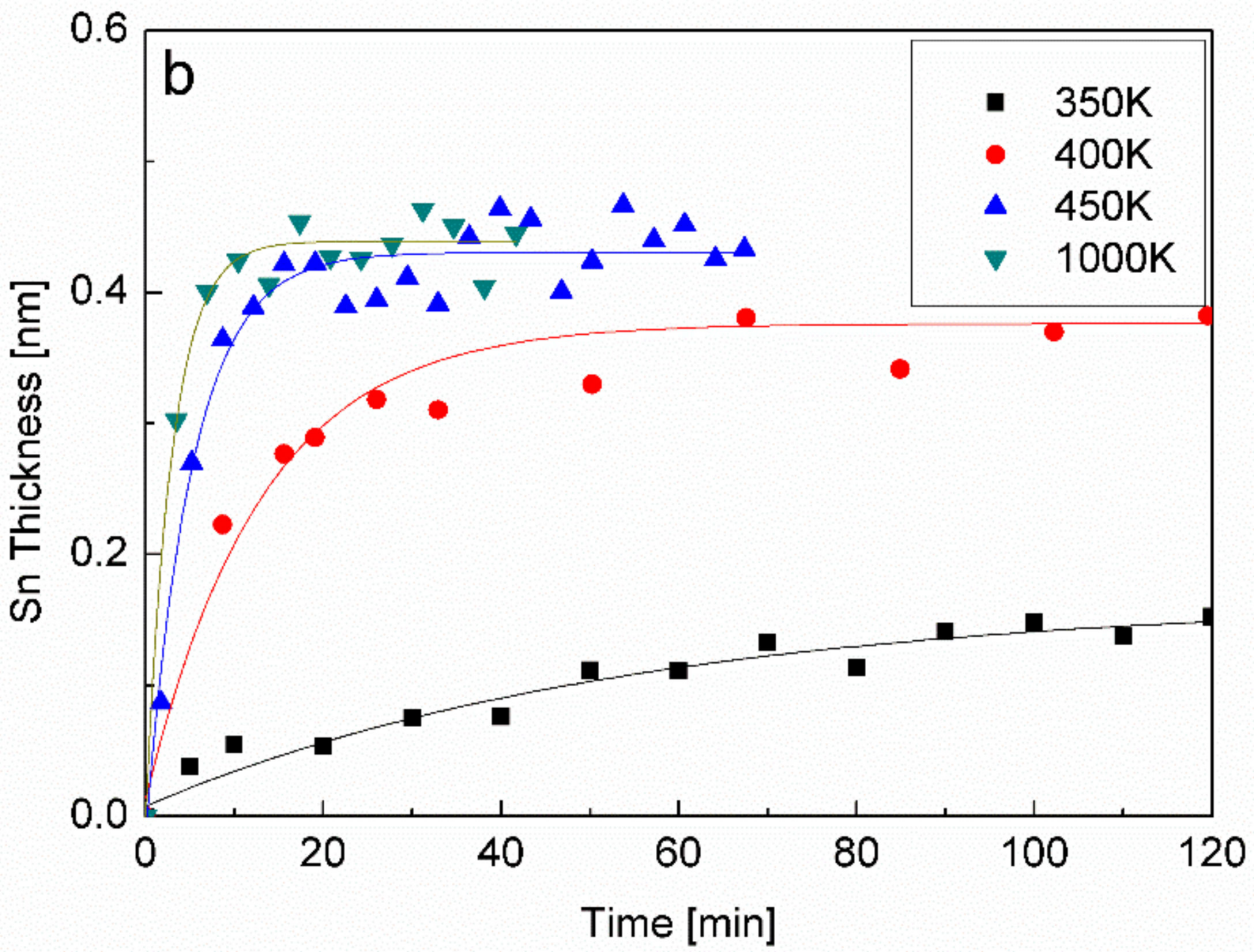
(**a**) Representative AES spectra of the alloy before and after heating the sample to 1000 K, indicating an attenuation of the Ti and Ni signals and an increase of the Sn; (**b**) Time depended Sn layer thickness (the lines are guide to the eye).

In order to evaluate the temperature dependence of the segregation, AES measurements were applied during the heating process (allowing much faster measurements relative to XPS). Figure 3 shows representative AES spectra taken before and after heating the sample to 1000 K and the formation of a Sn layer on the surface. Since the Ti and Ni signals were attenuated by the formation of the Sn overlayer, the layer thickness was estimated using the common formula shown in Equation (1) [26].

*d* = −λcosθ × ln(*I*/*I*_0_) (1)

here *d* is the overlayer thickness, λ is the inelastic mean free path (IMFP) of the electrons passing through the layer and *I* is the intensity of the underlying attenuated signal (the initial value is indicated by *I*_0_). θ is the collecting angle of the analyser (θ = 42° is the entrance angle of the electrons into the cylindrical mirror analyser (CMA) used for AES and θ = 0° for the hemispherical analyser used for XPS). The IMFP was evaluated using the NIST database (from the predictive formula by Gries) [27] and for Ti_LMM_ electrons (*E*_k_ = 387 eV), passing through the Sn layer, λ ≈ 0.8 nm. From Figure 3b it can be seen that Sn tends to segregate even at a low temperature such as 350 K, and at elevated temperatures the Sn layer on the surface reaches a thickness of ~0.4 nm, which is roughly about two monolayers. Although for most systems the tendency for segregation stops at one monolayer or less, it was found in multiple studies (mainly in the Fe-Sn system) that Sn tends to segregate for more than one monolayer and up to two monolayers on the surface [28,29,30,31,32], in agreement with the results found here. Similar results were also obtained by using the attenuation of the Ni signal and from the XPS spectra.

XPS measurements taken from the sputtered clean surface at RT revealed that the Ti 2p_3/2_ BE in the alloy is located at 454.2 ± 0.1 eV, ~0.3 eV higher than the literature value of 453.9 eV for pure Ti [20]. Similarly, the Ni 2p_3/2_ line measured for the alloy is located at BE of 852.9 ± 0.1 eV, ~0.3 eV higher than the value of 852.6 eV, known for pure nickel [19]. The BE of Sn 3d_5/2_ in the alloy was measured at 484.5 ± 0.1 eV, which is ~0.5 eV lower than the common value in the literature of 485 eV [21,23] and measured from a clean Sn sample on our instrument. These chemical shifts can be correlated to the bonding nature in the alloy. Senkovskiy et al. [33] investigated the electronic structure of Ti–Ni alloys by XPS and found that both of their BEs shift to higher values, up to 0.4 eV, compared to the pure elements. Since the core levels shifts were by a few tenths of eV to the same direction it was concluded that a charge transfer between Ti and Ni in the alloy is insignificant. In the case of the Ti–Ni-Sn alloy, opposite shifts are observed between both Ti and Ni compared to the Sn, implying on a charge transfer from the Ti and Ni towards the Sn. According to Graf et al. [4] in HH compounds, having the general formula XYZ, the X and Y atoms are characterized by a strong cationic behaviour and the Z atom exhibits an anionic one. In the TiNiSn case, X and Y are represented by Ni and Ti, respectively and Z atoms by Sn. This behaviour is consistent with the shift trend that was observed in the TiNiSn XPS measurements.

It is interesting to note, that after Sn segregation, which causes some Sn depletion in the near surface region, some shifts of ~0.2 eV towards lower BEs were measured for the Ti and Ni components and a shift of ~0.1–0.2 eV towards higher BE was measured for the Sn.

### 3.2. The Interaction with O_2_ at Room Temperature 

**Figure 4:**
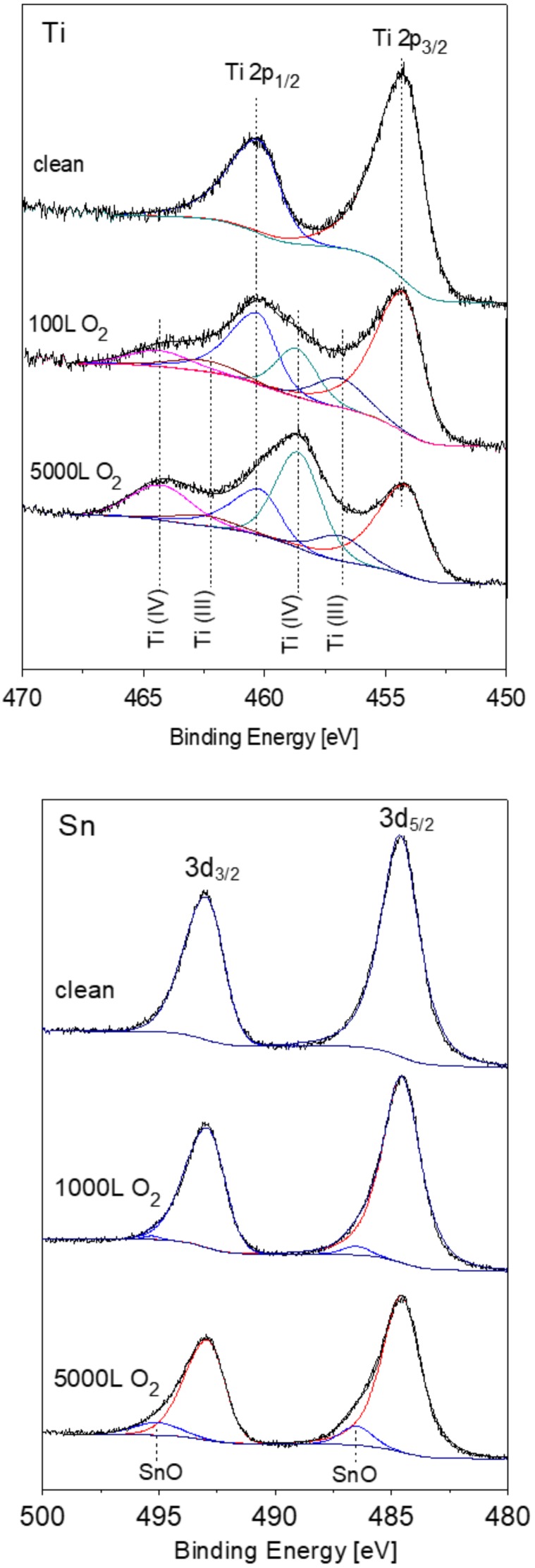
Representative XPS Ti 2p and Sn 3d spectra with the fitted metallic and oxide components.

XPS spectra of the alloy components were recorded following oxygen exposure at 1 × 10^−6^ Torr. Representative Ti 2p and Sn 3d spectra with the fitted metallic and oxidic components are depicted in Figure 4. It can be seen that the Ti component in the alloy is readily oxidized at RT and two oxidation states appear, Ti (III) which corresponds to Ti_2_O_3_ and Ti(IV) which corresponds to TiO_2_. In contrast to the Ti, only minor oxidation of Sn starts at high exposures, and no sign for Ni oxidation can be observed (spectra not shown).

**Figure 5:**
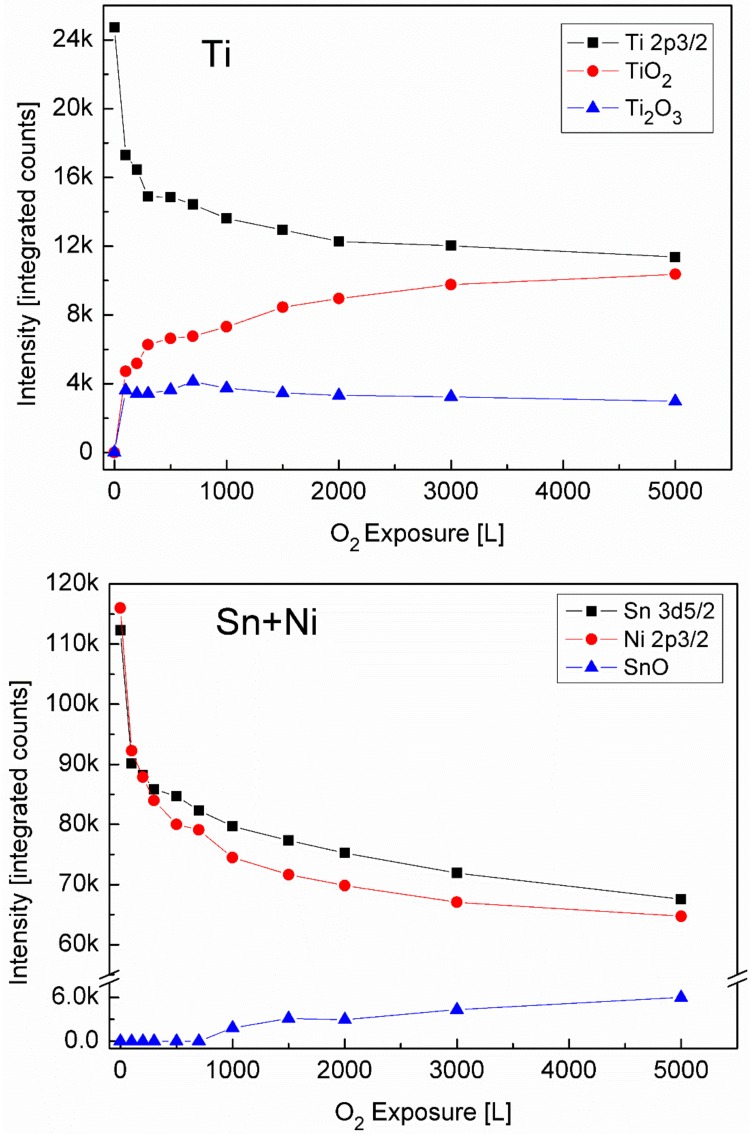
The metallic and oxides intensity of Ti, Ni and Sn vs. oxygen exposure at Room Temperature (RT).

The metallic and the oxidic fractions of the alloy components, as extracted from the XPS spectra are shown in Figure 5. It can be seen that the metallic Ti intensity decreases rapidly up to 100 L exposure and then its attenuation slows down. On the other hand, there is a fast increase in the Ti_2_O_3_ intensity (and the TiO_2_ one), that also continues to grow in relation to the metallic Ti decrease.

Observing the Sn and Ni lines, shows that they were attenuated during oxidation, while only the Sn shows some slight oxidation at 1000 L and above. This attenuation implies the possibility that some oxygen induced segregation of Ti to the surface occurs that screens the Sn and Ni signals, as is clearly seen at higher temperatures (Section 3.3). Since during oxidation of titanium (as well as other metals), the low oxidation states are located near the metal interface and the high oxidation states are located furthest from the metal (at the oxide–gas interface) [22], it can be deduced that an interface layer of Ti_2_O_3_ is formed on the surface and during the continuous oxidation, this layer’s outer side converts to TiO_2_.

An insight to the oxidation tendency of the alloy components can be gained by looking at the enthalpies of formation of the alloy constituents by oxygen [34]:

Ti + ^1^/_2_ O_2_(g) → TiO ΔH0 = −125 kcal/mol

2Ti + ^3^/_2_ O_2_(g) → Ti_2_O_3_ ΔH0 = −360 kcal/mol

Ti + O_2_(g) → TiO_2_ ΔH0 = −228 kcal/mol

Ni + ^1^/_2_ O_2_(g) → NiO ΔH0 = −57 kcal/mol

Sn + ^1^/_2_ O_2_(g) → SnO ΔH0 = −68 kcal/mol

According to the enthalpies of formation, titanium is considerably more reactive than Sn and Ni and, therefore, more susceptible to oxidation.

**Figure 6:**
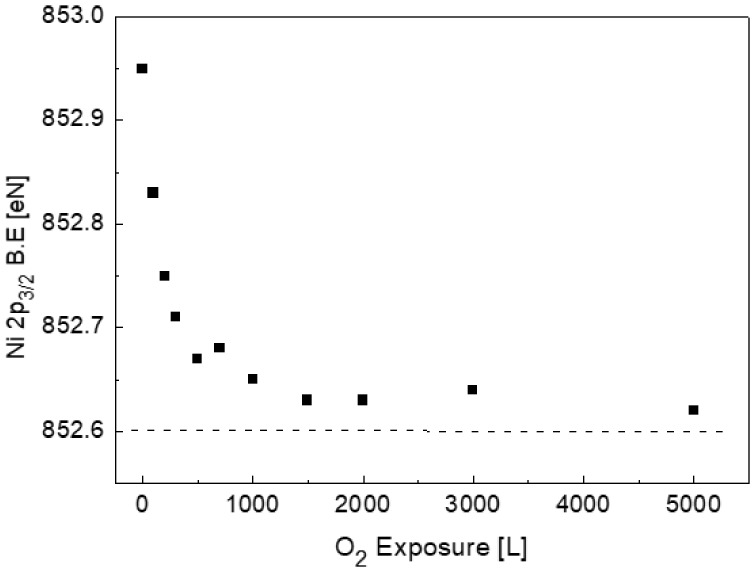
The change in the BE of Ni 2p_3/2_ vs. oxygen exposure. The BE decreases during oxygen exposure, approaching the value of pure Ni (the horizontal dashed line).

As mentioned in the previous paragraph, chemical shifts of ~0.3 eV towards a higher binding energy were measured for the Ti and Ni, and ~0.5 eV towards lower BE was measured for Sn (relative to the BE of the pure elements). It is, therefore, interesting to see whether the oxidation process, which mostly extracts the Ti atoms from the alloy bond, will influence these shifts. Figure 6 shows the change in the Ni 2p_3/2_ binding energy, as measured during the exposure to oxygen. Although no nickel oxidation was observed, a pronounced shift of its BE towards the known pure metallic value was measured, corresponding to the Ti oxidation. In contrast to the above, no significant shifts in the metallic Ti or Sn were measured relative to their location in the alloy.

### 3.3. The Interaction with O_2_ at 1000 K

**Figure 7:**
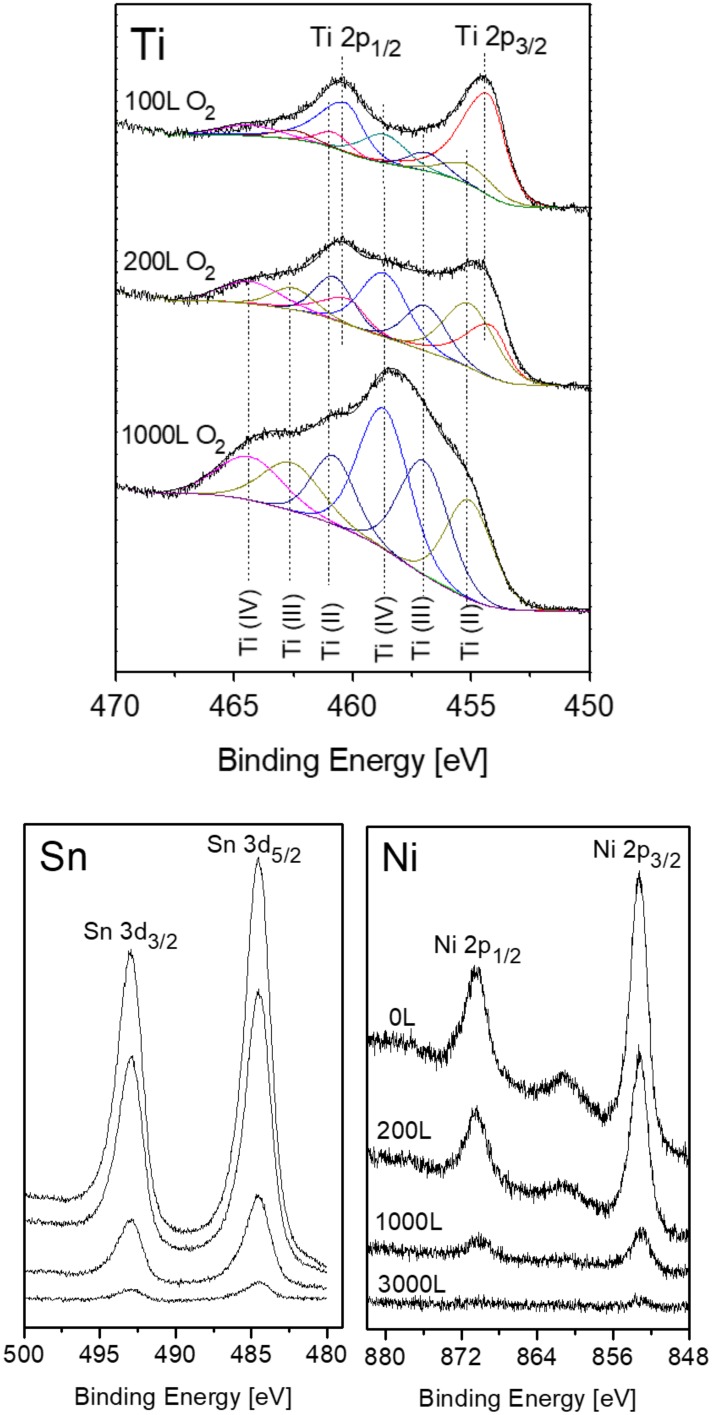
Representative XPS spectra of Ti, Ni and Sn after exposing to oxygen at 1000 K. Sn and Ni were exposed to identical amounts of oxygen. The order of the Sn spectra corresponds to the amount of oxygen exposure noted in the Ni spectra.

Prior to the exposure to oxygen at the elevated temperature, the sample was heated in vacuum at 1000 K for 10 min to allow Sn segregation to the surface. After each dose of oxygen, the oxygen was pumped out and the sample was cooled down to RT prior to XPS measurement. Representative XPS spectra of the alloy components during oxidation are presented in Figure 7. The spectra show rapid oxidation, a disappearance of the metallic Ti(0) signal and the formation of the Ti(II) oxidation state, that was not observed at RT. Similar to RT, only minor oxidation of the Sn and no oxidation of the Ni can be seen, but these signals attenuate significantly, until they totally disappear at higher exposures.

**Figure 8:**
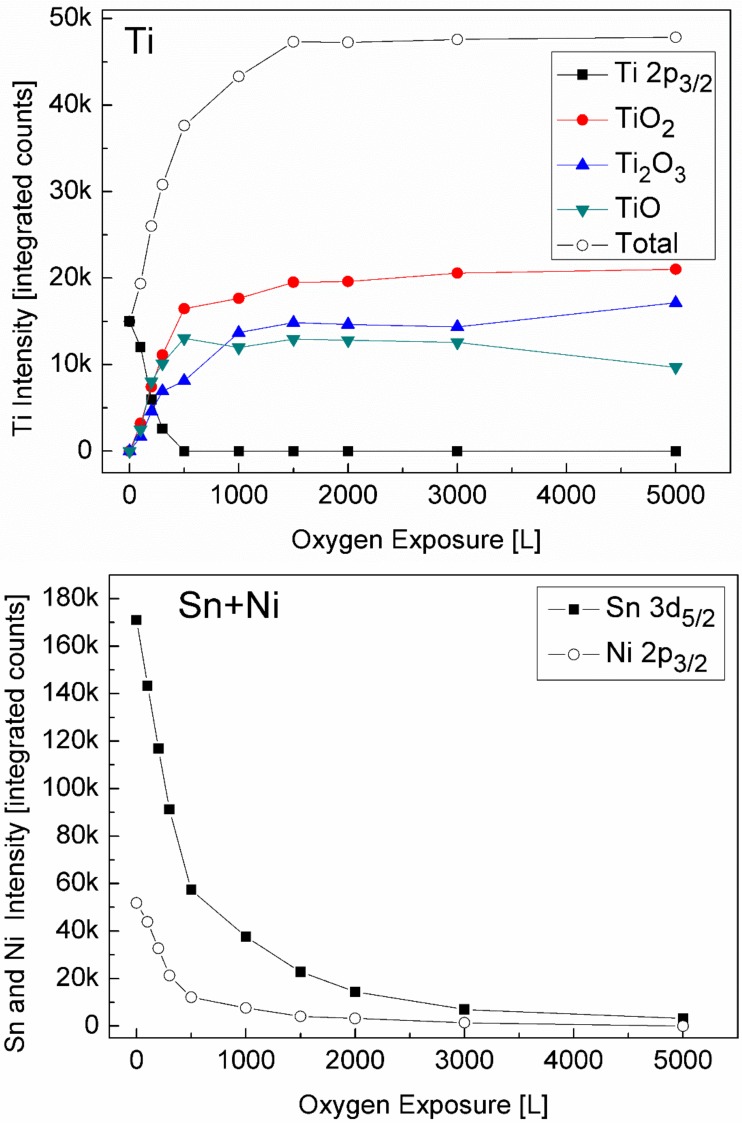
Intensity variation of the Ti and its oxides and of the Ni and Sn during oxygen exposure at 1000 K.

**Figure 9:**
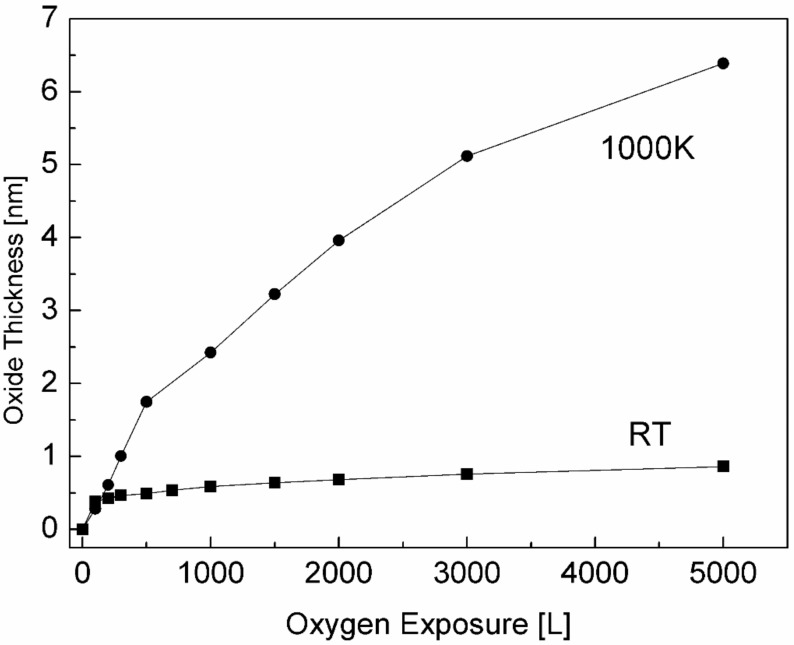
Oxide thickness formed on the surface during exposure to oxygen at RT and 1000 K.

The intensity of the Ti and its formed oxides and of the Sn and Ni are depicted in Figure 8. It clearly shows that at 1000 K, three oxidation states of the titanium exist and develop during oxidation; the TiO grows at the alloy–oxide interface, while TiO_2_ prevails on the outer surface, with Ti_2_O_3_ as an intermediate layer. The large increase in the total Ti signal and the simultaneity decrease in the Sn and Ni signals, until they disappear, imply that Ti from the alloy segregates to the surface (oxygen induced segregation) and that a titanium oxide layer is formed on top of the alloy (actually on top of the initial segregated Sn layer). The total titanium oxide thickness formed on the surface was estimated from the attenuation of the Sn signal according to Equation (1), and λ was evaluated as 1.6 nm [35]. The titanium oxide thicknesses formed on the surface at RT and 1000 K are presented in Figure 9.

**Figure 10:**
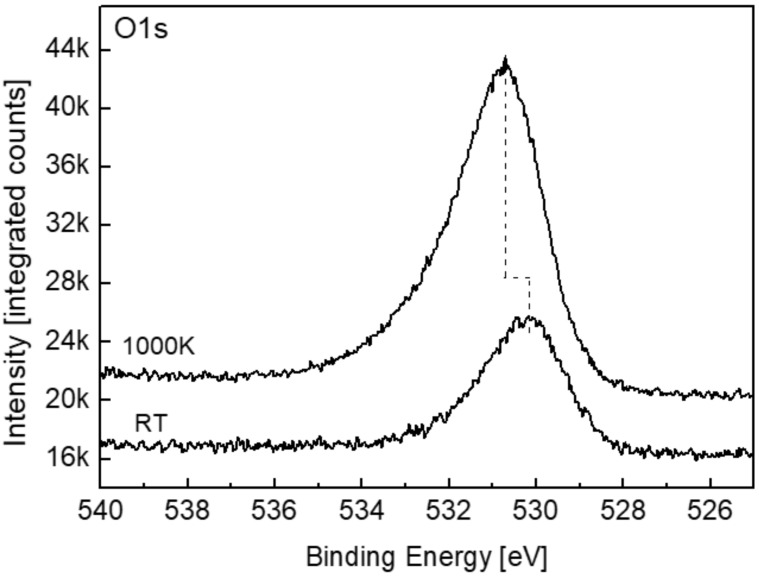
O1s spectra taken after exposure to 5000 L O_2_ at RT and 1000 K.

The O1s spectra (Figure 10), taken after 5000 L at RT and 1000 K, show some asymmetrical peaks, with BE that increase from 530.1 eV at RT up to 530.7 eV at 1000 K. The asymmetry probably stems from the variety of titanium oxides present, and the shift to higher energy at 1000 K is probably due to the more crystalline nature of the oxide formed at the elevated temperature and from the existence of TiO [22].

### 3.4. The Interaction with H_2_O at RT and 1000 K

**Figure 11:**
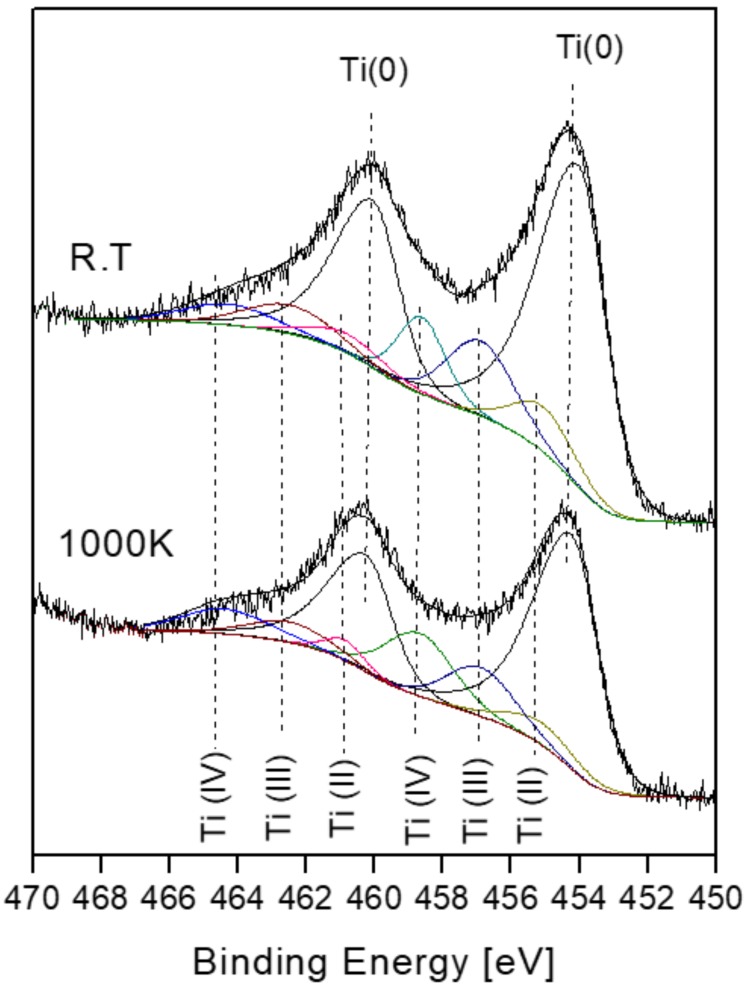
XPS spectra of Ti after exposing to 5000 L H_2_O at RT and 1000 K.

**Figure 12:**
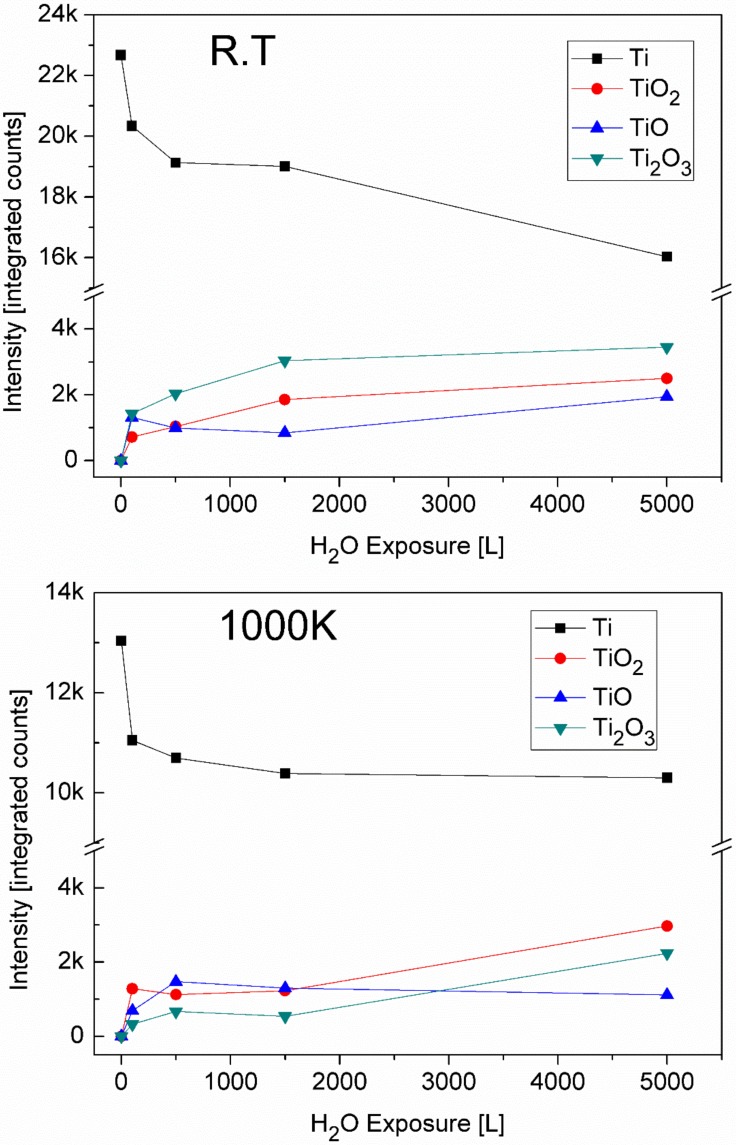
The change in the intensity of the Ti and its oxides during H_2_O exposure at RT and 1000 K.

As was seen in the previous paragraphs, the oxidation of the TiNiSn alloy during oxygen exposure is dominated by the reaction of titanium. In previous studies, it was found that titanium reacts much less with water vapor than with oxygen [22,36] and it is, therefore, intriguing to compare it to the alloy’s behaviour. Similar to the experiments with oxygen, the exposure at RT was performed on the sputter-cleaned alloy surface, while at 1000 K, the sample was first heated in vacuum to allow the Sn to segregate to the surface, before exposing to water vapor. The XPS spectra of Ti after exposing to 5000 L H_2_O at RT and 1000 K are presented in Figure 11. In contrast to oxygen exposure at RT, where only Ti(III) and Ti(IV) oxidation states were formed, during H_2_O exposure, there is also a formation of Ti(II) at RT, in agreement with Lu et al. [22], who reported that TiO is readily formed during water exposure. At 1000 K, there is only a minor decrease in the Ti(0) after water exposure, while after oxygen exposure, a thick oxide film forms on the surface and completely attenuates the metallic Ti after ~500 L. This behaviour is manifested in Figure 12, which shows the change in the intensity of Ti and its oxides. In addition, only relatively minor attenuation of the Sn and Ni were measured during exposure (data not shown), indicating a formation of a very thin oxide layer during water exposure, of ~0.5 nm, after 5000 L at RT and even less at 1000 K. It seems, therefore, that the segregated Sn layer acts somewhat as a barrier for oxidation by water vapor.

As shown above, the oxidizing ability of oxygen is considerably higher than that of water. This trend was also reported for other alloys [37,38] and is consistent with the more negative enthalpies of formation of metal oxides produced by the O_2_ reaction as compared to H_2_O, by a value of −56.9 kcal/mol, which refers to the formation of water [37].

## 4. Conclusions

In the present study, surface reactions of TiNiSn half-Heusler alloy were characterized at RT and during heating in vacuum, oxygen and water vapor up to 1000 K. The binding energies of Ti and Ni in the alloy exhibit a shift to higher values, while Sn was shifted to lower ones, compared to the pure elements. These chemical shifts can be correlated to the bonding nature in the alloy, where Ti and Ni are cations and Sn is an anion. During heating, Sn segregates to the surface of the alloy, forming an approximately two-monolayer thick layer. This segregation is probably driven by the tendency to lower the surface energy. On exposure to oxygen at RT, the main oxidative element was titanium with some minor oxidation of tin. Ti oxidized to TiO_2_ with some Ti_2_O_3_ formed at the interface with the metal. At 1000 K the exposure to oxygen promotes Ti segregation to the surface (oxygen induced segregation) and the formation of a titanium oxide layer, which completely attenuates the Ni and Sn XPS signals. The thickness of the oxide layer was evaluated as ~6.5 nm after 5000 L O_2_ exposure (compared to ~1 nm at RT) and it is composed of a mainly outer layer of TiO_2_ as well as Ti_2_O_3_ and TiO at the alloy interface. Water vapor was found to be a much milder oxidative gas compered to oxygen. The results shown here imply that the TiNiSn alloy may be susceptible to oxidation at high temperatures in oxygen or air and further work to investigate the kinetics and mechanism of oxidation under long term operation conditions has to be done.
